# Moderate and Severe Dental Fluorosis in the Rural Population of Anantapur, India: Change in Their Biological Susceptibility?

**DOI:** 10.3390/ijerph191811293

**Published:** 2022-09-08

**Authors:** Trina Mylena García-Escobar, Iván Valdivia-Gandur, Wilson Astudillo-Rozas, Oscar Aceituno-Antezana, Balasubbaiah Yamadala, Vicente Lozano de Luaces, Eduardo Chimenos-Küstner, María Cristina Manzanares-Céspedes

**Affiliations:** 1 Department of Odonto-Stomatology, Faculty of Medicine and Health Sciences, Universitat de Barcelona, 08007 Barcelona, Spain; 2 Biomedical Department, Universidad de Antofagasta, Antofagasta 1270300, Chile; 3 Dentistry Department, Universidad de Antofagasta, Antofagasta 1270300, Chile; 4 Biomedical Master of Science, Health Science Faculty, Universidad de Antofagasta, Antofagasta 1270300, Chile; 5 Kalyandurg Hospital, Anantapur 515761, India; 6 Rural Development Trust, Vicente Ferrer Foundation, 08029 Barcelona, Spain; 7 Human Anatomy and Embryology Unit, Faculty of Medicine and Health Sciences, Universitat de Barcelona, 08007 Barcelona, Spain; 8 UNIPRO—Unidade de Investigação em Patologia e Reabilitação Oral, Instituto Universitário de Ciências da Saúde (IUCS), CESPU, 4585-116 Gandra, Portugal

**Keywords:** oral health, fluorosis, quality of life, fluorosis prevalence

## Abstract

Dental fluorosis affects the quality of life. A cross-sectional, observational study was conducted in a community affected by endemic fluorosis for several generations with a conserved biological and social environment. The study included patients from the rural population of Anantapur, India. The Dean index (DI) and the Thylstrup and Fejerskov Index (TFI) were used for fluorosis classification. Additionally, water samples were collected for fluoride analysis, taken from the patients’ living areas. The statistical association between the variables was analyzed. In total, 785 patients between 10 and 60 years old were included in the study (58.7% women and 41.3% men). Fluorosis signs were found in 94.6% of patients examined using the DI and 94.4% using the TFI. Moderate–severe dental fluorosis was observed in 62.8% by DI and 73.1% by TFI consuming untreated water with up to 2.9 ppm of fluoride. Furthermore, moderate–severe dental fluorosis was observed in 33.2% by DI and 39.9% by TFI consuming water with ≤1.5 ppm of fluoride. The high prevalence of moderate–severe dental fluorosis in patients consuming water with a low fluoride concentration suggests that other factors are involved. Biological susceptibility change could play an essential role in the severity of dental fluorosis in populations exposed for several generations, affecting its actual and future quality of life.

## 1. Introduction

Organic and inorganic fluorides are present in nature from various sources. Inorganic fluorides are particularly relevant because of their effect on human health [1,2]. In the crust of the earth, fluorides are found in different concentrations depending on the geological environment. In this regard, the World Health Organization (WHO) has identified areas in the five continents that present high amounts of fluorides [3,4,5]. Water in its cycle must pass through the deeper underground layers, being thus affected by natural fluoride pollution [6]. For this reason, the literature has described the presence of endemic fluorosis in certain geographic areas whose population uses groundwater for drinking. Human consumption of water constitutes the major mechanism of fluoride incorporation [6]. Exposed populations in areas of endemic fluorosis have evidenced skeletal and dental fluorosis for several generations, due to chronic exposure to high amounts of fluoride. Additionally, other factors are involved in dental fluorosis including the population’s nutrition, weather conditions, individual susceptibility, biological response, and genetic influence [7,8,9,10,11,12].

In India, fluorosis is endemic in at least 20 states [13,14]. The principal source of drinking water is groundwater for rural populations [15], fluoride concentration levels varying depending on geographic localization. Although several studies examined the problem of fluorosis in Indian states [16,17], there is a need for prevalence studies in specific regions whose populations share the sociocultural environment as well as weather and geography, in order to generate relevant information about the long-term effects of the fluoride ingested for several generations. Anantapur is a district whose population is essentially rural (71.93% according to the 2011 census), making most of their living from agriculture and consuming groundwater as their main source of drinking water. Similar to other Indian rural regions, Anantapur (from Andhra Pradesh State, India) has a complex social structure where factors such as the caste system, interpopulation segregation, inbreeding, or lack of mobility [16,17] have been observed for several generations and are still detected, although the Government of India policies have established strategies to improve these aspects. 

The hypothesis was that the chronic intake of a high amount of fluoride for several generations can alter the biological response and the physical manifestations of dental fluorosis, in a population whose socio-cultural environment has not experienced significant changes over time, affecting the actual and future quality of life. The aim of this study is to describe the prevalence of severe dental fluorosis in patients from a rural community of the Anantapur district, southwest of the Andhra Pradesh state and its relation to the fluoride content of the drinking water. 

## 2. Materials and Method

### 2.1. Study Characteristics, Population, and Ethical Aspects

**Figure 1:**
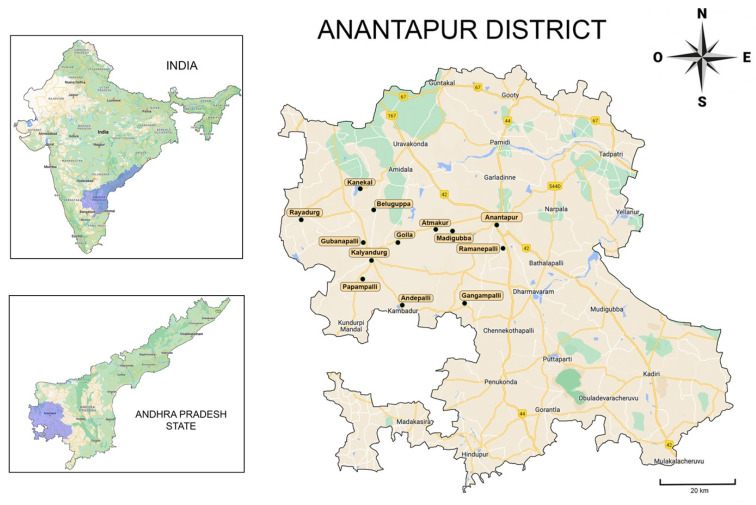
The 13 rural communities of Anantapur visited by the dental brigades included in the study (remarked). Images modified from Google Maps.

The study is cross-sectional and observational. The sample for this study was non-probabilistic and included subjects from 13 rural communities of Anantapur district, state of Andhra Pradesh, India (Figure 1). The director of the Kalyandurg Hospital and coordinator of the Rural Development Trust Kalyandurg-Kanekal, who represents the health authority of the region, authorized the study and participated in the elaboration of the instruments to obtain the study data. The instruments for collection and management of clinical data and written informed consents were revised and approved by the Bioethics Committee of the University of Barcelona (number 3-2011) and performed according to the Helsinki declaration. The study was conducted during dental care activities of the rural brigades under the direction of the Kalyandurg Hospital and Vicente Ferrer Foundation. Every year, oral health professionals perform oral health promotion activities involving prevention, prophylaxis, and dental treatments when required. The activities are offered to the population, but the patient decides voluntarily to receive them. When a patient met the inclusion criteria, their data were obtained after a detailed verbal explanation of the study process and objectives (with the help of translators), and written informed consent was solicited. Patients under 18 years were asked for their consent for the examination and additionally, written informed consent was solicited from their parents or legal tutors. During the clinical interview, the patients were asked about the water source they used for drinking. Once this information was obtained, the oral examination was carried out, including registries of the fluorosis signs and clinical photography, safeguarding the identity of the patients.

### 2.2. Inclusion/Exclusion Criteria

The inclusion criteria were:

- Patients of both genders.
- Patients born in the rural community of Anantapur.
- Patients with at least two teeth in each dental group, without dental destruction due to caries or trauma (independent of age).
- Patients who declared to consume water from untreated sources (groundwater).
- The exclusion criteria were:
- Patients with orofacial malformations or pathologies that could alter or increase the difficulty of the examination.
- Patients with systemic pathology affecting fluoride metabolism.
- Patients without permanent or definitive teeth.
- Patients with dental surface wear or stains due to tobacco, betel, or another chewing habit, impairing an adequate dental examination.
- Patients with an excess of bacterial dental plaque or calculus impairing an adequate dental examination.
- Patients requiring urgent dental attention.
- Patients who did not answer all the questions and those from whom it was difficult to obtain valid information.
- Patients whose parents or grandparents came from another community outside Anantapur.

### 2.3. Indices Used for Dental Fluorosis Classifications

For the classification of dental fluorosis, two indices for fluorosis prevalence were applied to all patients. The Dean index (DI) was used according to WHO guidelines [18]. The DI considers six scores to assess the damage to the tooth surface, from 0 (normal) to 5 (severe). The Thylstrup and Fejerskov Index (TFI) was also used. The TFI uses an ordinal scale from 0 to 9, where 9 depicts the most serious effects [19]. Moreover, the TFI offers a subclassification of the severe form of dental fluorosis. Four examiners were separated into two teams, assigned by the letters A and B. Team A (two dentists) was calibrated to use the DI criteria and Team B (two dentists) was calibrated to use the TFI. The calibration was performed using the agreed-upon criteria by the Forum on Fluoridation 2002 [20] and relevant literature [18,19]. The Kendall coefficient of concordance (KCC) was used to assess the interobserver agreement. During the training time, each team applied the DI or TFI for fluorosis evaluation considering a group of 25 patients. These tests were repeated after 5 and 10 days. The interobserver KCCs of Team A (DI) varied between 0.756 and 0.874 while Team B (TFI) varied between 0.689 and 0.831. Both teams examined all the patients that meet the inclusion/exclusion criteria for the study.

### 2.4. Collection and Analysis of Water Samples

Drinking water samples were taken from the sources available for patients in rural communities from Anantapur. The source location was obtained from the information provided by patients during the compilation of general antecedents. Three samples for each source were taken on different days and stored in sterile polystyrene tubes (50 mL). The average concentration of fluorides obtained from the three samples was assigned to the source as a variable. If a community had more than one source, three samples were taken from each one and the average fluoride concentration was assigned to the rural community. The analysis of the fluoride content in the samples was conducted in the laboratory Labaqua Catalunya (Barcelona, Spain), using ion chromatography according to the parameters for potable waters for public consumption in Spain (R.D. 140/2003).

### 2.5. Statistical Analysis

First, the fluorosis values considering the DI were dichotomized into “Normal + Questionable–Very mild–Mild” (Normal + QVM) and “Moderate–Severe” (MS) groups, while the TFI was also dichotomized into “Normal + 1–3” and “49”. According to equivalency criteria between the DI and TFI accepted in the literature, the DI “Normal+QVM” group corresponded to TFI 1–3, while the DI “MS” group corresponded to TFI 4–9 [21]. After the normality test (with the Kolmogorov–Smirnov test), Mann Whitney U and Kruskal–Wallis tests (including the Mann Whitney U as the post-hoc test) were applied to compare independent samples. Additionally, the Pearson Chi-square test, Fisher exact test, and odds ratio were used to establish the independence or association between dichotomized fluorosis indices with age range, gender, and fluoride concentration in water. Age-range was divided into six groups (up to 15, 16–25, 26–35, 36–45, 46–55, and over 55 years), while fluoride concentration levels found in the water samples were divided into two groups according to the WHO indication about fluoride level in drinking water, where values ≤1.5 ppm are considered acceptable and over 1.5 are not [5]. Spearman’s Rank Order was applied to analyze the correlation between the concentration of fluoride in the water detected and the percentage of fluorosis MS (DI) and 4–9 (TFI) observed in each community. SPSS 25 (Chicago, IL, USA) and G*Power Version 3.1.9.6, Heinrich-Heine-Universität Düsseldorf, Düsseldorf, Germany (post-hoc test) were used for statistical analysis. The level of significance considered was *p* < 0.05. 

## 3. Results

### 3.1. General Characteristics of the Studied Population and Water Samples

In total, 1142 patients were examined, of which 785 satisfied all the inclusion criteria, with 463 women (58.7%) and 322 men (41.3%) from 13 rural communities (Figure 1, Table 1). The average age was 22.84 for women (SD 12.92) and 24.22 for men (SD 13.14), with no statistical differences between genders (*p* = 0.123), showing a high concentration of young population. However, significant differences were observed in the variable age among rural communities (*p* = 0.000). The post-hoc test shows that the communities of Kalyandurg and Papampalli had the youngest population in the study. Additionally, a significant association was observed between the variable gender and the variable community (*p* = 0.002), indicating a heterogeneous distribution of women and men in the communities included in the study. 

The values of fluoride concentration in waters obtained showed a range from 1.1 to 2.92 ppm (average 1.71, median 1.5). Table 1 shows the patient’s demographic data concerning this study, the fluoride values observed in drinking water considering each community studied, and the distribution of fluorosis cases diagnosed considering the dichotomized DI and TFI indices. 

### 3.2. Dental Fluorosis Prevalence and Association with the Variables Studied

**Figure 2:**
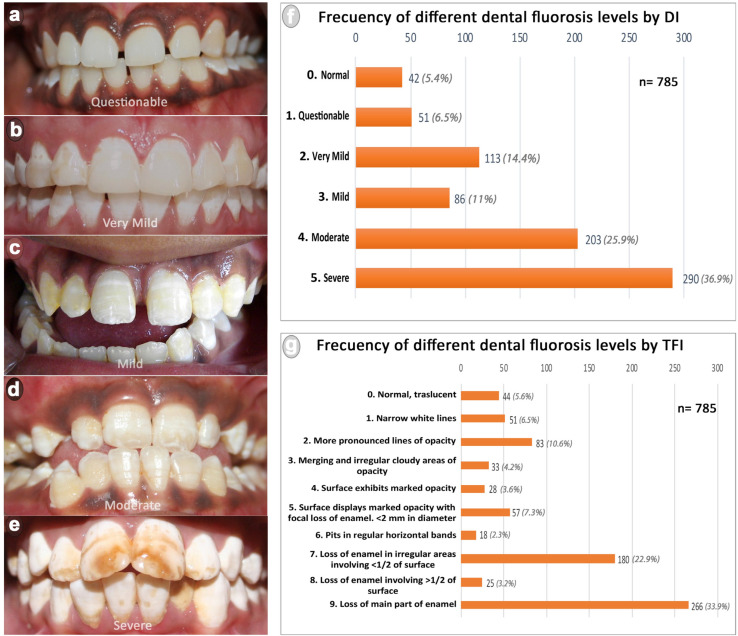
Frequency of different dental fluorosis levels observed in the rural populations of Anantapur. (**a**–**e**) show fluorosis levels considering the DI (from questionable to severe); (**f**) frequency of dental fluorosis by DI; (**g**) frequency of dental fluorosis by TFI.

A general prevalence of 94.6% of dental fluorosis was observed by Team A (DI), while Team B (TFI) detected a 94.4% prevalence. Distribution and clinical aspects of patients considering fluorosis indices levels are presented in Figure 2. Concerning the dichotomized indices, a high prevalence of MS cases by DI (62.8%) and 4–9 cases by TFI (73.1%) were observed, evidencing a predominance of the most severe forms of fluorosis in the population studied. Additionally, a significant association between fluorosis MS by DI and 4–9 by TFI with young patients was observed (*p* value = 0.000). Considering the fluoride concentration in the drinking water, 54.3% of patients examined with the DI and 54.5% examined with the TFI showed dental fluorosis consuming water with ≤1.5 ppm of fluoride. On the other hand, 33.2% of the patients examined with the DI showed dental fluorosis MS and 39.9% examined with the TFI showed dental fluorosis 4–9 while consuming water with ≤1.5 ppm of fluoride. However, a significant association was observed between patients showing fluorosis MS by DI or 4–9 by TFI with the consumption of >1.5 ppm of fluoride in drinking water. The odds ratio shows that the patients who consumed water with > 1.5 ppm of fluoride were 1.81 times more likely to have fluorosis MS by DI and were 1.79 times more likely to have fluorosis 4–9 by TFI, compared with patients who consumed water with ≤1.5 ppm of fluoride. No association was observed between the variable gender and the dichotomized level of fluorosis. However, a significant association was observed between 36–45-year-old women and fluorosis MS by DI, and 4–9 by TFI. The odds ratio evidenced that women between 36 and 45 years old were 3.07 times more likely to have fluorosis MS by DI and 2.83 times more likely to have fluorosis 4–9 by TFI than men. Additionally, the odds ratio showed that the females who consumed >1.5 fluorides in drinking water were 1.74 times more likely to have fluorosis 4–9 (TFI) than men. Table 2 presents the association between dichotomized indices and the variables studied.

**Figure 3:**
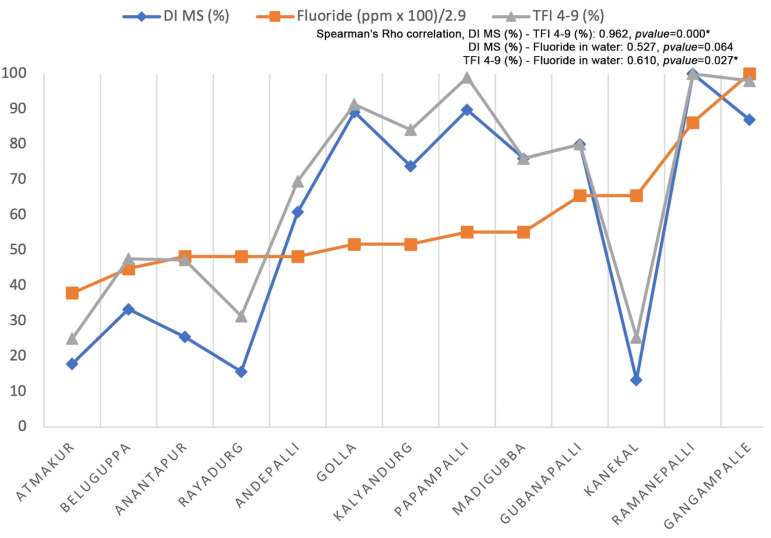
Spearman’s Rho correlation considering fluoride concentration found in water samples obtained in each community included in the study and the percentage of cases considered MS by DI and 4–9 by TFI observed in each community. The major value of fluoride observed in drinking water is 2.9. * Significant correlation.

The Spearman correlation (Figure 3) showed a significant correlation between MS (DI) and 4–9 (TFI) values (0.962, *p* value = 0.000) and between fluoride concentration in drinking water with 4–9 (TFI) values (0.610, *p* value = 0.027). 

## 4. Discussion

### 4.1. The Situation of Anantapur District, Andhra Pradesh State

Endemic problems caused mainly by excessive fluoride in the groundwater have been reported in the state of Andhra Pradesh [3,22]. The 2011 official data of the Indian census [23] showed that, overall, 37.3% of Anantapur rural households use water from treated sources, while 62.7% use untreated water from several sources, such as groundwater from covered or uncovered wells, rivers or canals, cisterns, ponds, or lakes. In this study, all patients reported consuming water from untreated groundwater. A report generated by the Indian government expert committee described that 85% of the rural population from Andhra Pradesh use groundwater for drinking and domestic purposes, with values of fluoride over 1.5 ppm [24]. Anantapur is an arid region, as indicated by the official website of the Anantapur district [25], and the rainfall is not only scanty but also erratic in nature [6], decreasing its capacity to dilute the fluoride amount in the groundwater. The WHO general recommendation indicates that regions such as Anantapur with high temperatures (over 20 degrees Celsius during the year) must maintain concentrations of fluoride in the drinking water between 0.5 and 0.7 ppm, due to the high water consumption caused by their type of geological environment and weather conditions [26]. Given the characteristics of the region, the water samples included in the study (Table 1) revealed levels above 1.0 ppm of fluoride in all sources, with a wide margin of variation between communities. However, the fluoride levels found in drinking water seem insufficient to explain the high prevalence and severity of dental fluorosis observed.

### 4.2. Dental Fluorosis in the Studied Population and Its Relation to the Regional Drinking Water

Frequently, the literature deals with the effect of fluoride toxicity in oral health considering a specific period of life; for example, if the exposition occurs during the enamel formation and mineralization process, the damage to the teeth will be more severe [27,28], including adverse changes in the chemical composition of tooth enamel and its structure [29]. In addition, childhood implies a natural greater risk for the toxic effects of fluorides compared to adults because children have greater metabolic rates of fluoride retention due to the natural incorporation of the fluoride to the growing skeletal and dental structures [30]. A similar effect has also been observed in women during pregnancy [31]. Consequently, there is little focus on the long-term effect of fluoride toxicity on human biology or the effect of this toxicity for several generations in communities such as Anantapur. The study performed here observed a high prevalence of dental fluorosis (94.6% by DI and 94.4% by TFI) with a predominance of moderate to severe cases (62.8% by DI and 73.1% by TFI) in a population consuming water with up to 2.9 ppm of fluoride in drinking water (average 1.7). In general terms, these results are moderately consistent with similar studies conducted in Anantapur and other Andhra Pradesh districts. For example, a previous study carried out in Andhra Pradesh state concerning 6586 villages from 21 districts revealed that Anantapur was the district with most villages affected by dental fluorosis, considering levels of fluoride in drinking water from 1.52 to 4.45 ppm (average 1.99) [32]; in another study in a child population of Anantapur, Nalgonda and Khammam showed a wide difference in the prevalence of dental fluorosis that varied from 38.4 to 100%, with 5.7 ppm being the maximum concentration of fluoride found [33,34,35,36,37]. It is important to note that the study performed here observed 33.2% and 39.9% of moderate–severe dental fluorosis cases (MS by DI and 4–9 by TFI, respectively) considering up to 1.5 ppm of fluorides in drinking waters. In this regard, the literature shows diverse and controversial information. For example, in the Khammam district, a 17.65% prevalence of the moderate–severe form of dental fluorosis was observed, while the fluoride content in water was up to 3.5 ppm [37]; another study in Mysore, Karnataka, India, showed 17.65% of moderate–severe dental fluorosis considering up to 1.2 ppm of fluoride concentration in drinking water [38]; in Bagalkot district, Karnataka, India, 100% of moderate–severe dental fluorosis prevalence was reported with 1.36 ppm of fluoride in drinking water, considering 93 individuals aged 9–15 years. [39]. Along the same lines, wide differences in results are also seen in studies conducted outside of India. For example, in Saudi Arabia, the prevalence of moderate–severe fluorosis in a child population of 12 regions varied from 7.32% to 41.25%, considering a fluoride concentration in drinking water up to 1.69 ppm [40,41]; in the Hidalgo state, Mexico, the prevalence of the moderate–severe form of fluorosis was observed in 76.3% consuming drinking water with a 2.4–3.2 ppm range of fluoride [42]; in the Kiambu district, Kenya, 92% of the moderate–severe form of dental fluorosis was reported in the child population, considering fluoride in drinking water up to 2.1 ppm [43]; another study in two villages from Khartoum, Sudan, with values of fluorides in drinking water up to 2.56 ppm, showed a prevalence of 31.8% of moderate–severe dental fluorosis in a child population [44]. As it is possible to observe, the articles reviewed about the prevalence of dental fluorosis report differences concerning inclusion criteria, data presentation, range of fluoride concentration in water considered acceptable, the method used for fluorosis diagnosis, among others, which makes the comparative analysis with the results described here quite difficult. It is worth mentioning that these studies have not considered other fluoride sources in the analysis (e.g., food). In this regard, fluoride concentration in different foods has been studied in the Anantapur population, showing that the quantification of fluoride intake can increase significantly when foods are considered [45]. However, the quantitative determination of fluoride intake by the child population is very difficult in their first years of life, when fluorotic changes in enamel are more severe if the secretory ameloblast and the maturation stage of enamel are exposed to critical fluoride levels chronically [46]. 

### 4.3. Polymorphism, Epigenetic and Genotoxicity Associated to Fluoride: Potential Changes in the Biological Susceptibility of the Organism against Fluoride as a Toxin

The rural population of Anantapur could be expressing an intrinsic biological change due to the chronic water consumption with critical concentrations of fluoride throughout people’s lives and for several generations. Additionally, intrinsic aspects of the population can also contribute to the phenomenon, such as its complex nuclear structure, scarce mobility, and low genetic variability [47,48]. In this regard, genetic variations and alterations can change the biological response of an organism against fluoride as a toxin. For example, different dental fluorosis manifestations have been related to ethnicity [49,50]; additionally, an association was observed between dental fluorosis and the polymorphism of the genes COL1a2 (collagen type 1) [11,51], TIMP1 (metalloproteinase inhibitor), DLX1 and 2 (homeobox transcription factor genes, associated with craniofacial development) [52], ESR (estrogen receptor) [53,54], and CTR (calcitonin receptor) [55]; specifically in India, associations between dental fluorosis and polymorphisms of the genes COL1a2 [56,57,58], ESR [58], BGLAP [58] (a protein that regulates bone remodeling), and SPARC [58] (protein required for the bone calcification process) have been evidenced; additionally, the polymorphisms of both DLX 1 and 2 genes (craniofacial development) were associated with the risk of severe fluorosis [52]. In animal models, it has been shown that both the genetic and environmental factors play a role in tooth quality [59,60]. On the other hand, genotoxicity induced by high doses of fluorides has been observed in in vitro and in vivo models [61], affecting bone and ameloblast cell lines. For example, osteosarcoma cell lines exposed to doses of 8 ppm of NaF showed alterations in the expression of genes associated with bone formation [62]; another study in male mice showed gaps and fissures of chromatids in the metaphase stage after exposition to high fluorine concentrations (4–20 mg/L) [63]; additionally, when the cell was exposed to concentrations of up to 5 mM of sodium fluoride, phosphorylation of the histone H2AX of LS8 ameloblast DNA was observed, which can induce cell apoptosis [64,65]. Finally, alterations in gene expression resulting from exposure to a toxin can be transmitted to subsequent generations through transgenerational epigenetic inheritance [66,67]. This phenomenon suggests that chronic exposure to fluorides could trigger heritable epigenetic changes that alter the biological response against fluoride as a toxin.

As the main limitation of this study, the dental brigades of the Vicente Ferrer Foundation carry out prevention and treatments when needed by underserved populations. Therefore, although several inclusion/exclusion criteria were applied, the interpretation of the results should be performed with caution, because the sample included volunteer patients with different treatment needs and in a specific time. Randomized stratified studies are needed to complement and expand the results reported in our study. In addition, other aspects related to the quality of drinking water should be included in future studies, such as pH or the presence of other minerals.

## 5. Conclusions

Patients from rural communities of the Anantapur district showed a high prevalence (over 90%) of dental fluorosis. Moreover, the Anantapur population presents a high number of moderate and severe cases (over 60%), while other populations showed less severe forms of fluorosis, despite reporting superior fluoride levels to those found in the Anantapur drinking water. The severity of fluorosis concerning fluoride concentration levels in drinking water in Anantapur suggests that other factors are involved in the severity of the dental fluorosis observed. A potential change in the biological susceptibility of the population to the toxin, due to the long-term exposition (including several generations) could explain the phenomenon, affecting its actual and future quality of life. 

## Figures and Tables

**Table 1:** Rural population distribution, fluoride quantity in water, and fluorosis index observed considering the Dean index (DI) and the Thylstrup and Fejerskov index (TFI) N = 785.

Rural Community	Patients Included	Female	Male	Age Mean (Range)	Drinking Water Sources Analyzed	Fluoride Quantity in Water (ppm)	DI Normal + QVM *(a)*	DI MS *(b)*	TFI Normal + 1–3 *(c)*	TFI 4–9 *(d)*
Anantapur	55	35	20	27.5 (10–60)	1	1.4	41	14	29	26
Andepalli	23	19	4	18.5 (10–40)	1	1.4	9	14	7	16
Atmakur	28	18	10	28.8 (16–51)	1	1.1	23	5	21	7
Beluguppa	21	16	5	35.1 (11–60)	1	1.3	14	7	11	10
Gangampalli	100	46	54	29.9 (10–60)	1	2.9	13	87	2	98
Golla	46	26	20	25.6 (10–60)	1	1.5	5	41	4	42
Gubanapalli	10	6	4	24.7 (12–55)	1	1.9	2	8	2	8
Kalyandurg	233	149	84	19.2 (10–60)	2	1.5	61	172	37	196
Kanekal	75	31	44	28.0 (10–55)	1	1.9	65	10	56	19
Madigubba	25	12	13	25.8 (11–55)	1	1.6	6	19	6	19
Papampalli	98	63	35	13.1 (11–20)	2	1.6	10	88	1	97
Ramanepalli	20	10	10	27.5 (16.50)	1	2.5	0	20	0	20
Rayadurg	51	32	19	27.9 (10–60)	1	1.4	43	8	35	16

*(a)* Dean index normal–questionable–very mild-mild; *(b)* Dean index moderate–severe. *(c)* Thylstrup and Fejerskov Index normal plus levels 1, 2, and 3. *(d)* Thylstrup and Fejerskov Index levels 4–9. For more detail see Section 2.5.

**Table 2:** Analysis of fluorosis distribution considering gender, age range, and fluoride concentration in water observed.

		Dean Index *(a)* n = 785					Thylstrup Fejerskov Index *(a)* n = 785				
		MS (%)	Normal + QVM (%)	*p* Value	SP	OR (CI 95%) *(d)*	4–9 (%)	Normal + 1–3 (%)	*p* Value	SP	OR (CI 95%) *(d)*
**Age range (y = years)**											
up to 15 y		269 (34.2)	47 (5.9)	0.000 * (b)	1.0		295 (37.5)	21 (2.6)	0.000 * (b)	1.0	
16–25 y		103 (13.1)	97 (12.3)				131 (16.6)	69 (8.7)			
26–35 y		51 (6.4)	78 (9.9)				66 (8.4)	63 (8.0)			
36–45 y		41 (5.2)	36 (4.5)				46 (5.8)	31 (3.9)			
46–55 y		23 (2.9)	21 (2.6)				28 (3.5)	16 (2.0)			
over 55 y		6 (0.7)	13 (1.6)				8 (1.0)	11 (1.4)			
**Fluoride concentration**											
more than 1.5 ppm		232 (29.5)	96 (12.2)	0.000 * *(c)*	0.97	1.81 (1.34–2.45) *	261 (33.2)	67 (8.5)	0.000 *(c)	0.94	1.79 (1.28–2.5) *
up to 1.5 ppm		261 (33.2)	196 (24.9)				313 (39.8)	144 (18.3)			
**Gender**											
female		294 (37.4)	169 (21.5)	0.653 *(c)*		1.08 (0.8–1.44)	350 (44.5)	113 (14.3)	0.07 *(c)*		1.35 (0.98–1.86)
male		199 (25.3)	123 (15.6)				224 (28.5)	98 (12.4)			
**Age range considering gender (y = years)**											
up to 15 y	female	166 (52.5)	30 (9.4)	0.871 *(c)*		0.91 (0.48–1.74)	187 (59.1)	9 (2.8)	0.067 *(c)*		2.31 (0.94–5.66)
	male	103 (32.5)	17 (5.3)				108 (34.1)	12 (3.7)			
16–25 y	female	57 (28.5)	59 (29.5)	0.475 *(c)*		0.8 (0.45–1.4)	76 (38)	40 (20)	1.000 *(c)*		1 (0.55–1.81)
	male	46 (23)	38 (19)				55 (27.5)	29 (14.5)			
26–35 y	female	31 (24)	44 (34.1)	0.716 *(c)*		1.2 (0.58–2.46)	39 (30.2)	36 (27.9)	0.860 *(c)*		1.08 (0.54–2.18)
	male	20 (15.5)	34 (26.3)				27 (20.9)	27 (20.9)			
36–45 y	female	26 (33.7)	13 (16.8)	0.023 * *(c)*	1.0	3.07 (1.21–7.28) *	28 (36.3)	11 (14.2)	0.038 * *(c)*	0.99	2.83 (1.1–7.27) *
	male	15 (19.4)	23 (29.8)				18 (23.3)	20 (25.9)			
46–55 y	female	12 (27.2)	15 (34)	0.228 *(c)*		0.44 (0.12–1.52)	17 (38.6)	10 (22.7)	1.000 *(c)*		0.93 (0.26–3.28)
	male	11 (25)	6 (13.6)				11 (25)	6 (13.6)			
over 55 y	female	2 (10.5)	8 (42.1)	0.350 *(c)*		0.31 (0.04–2.38)	3 (15.7)	7 (36.8)	0.370 *(c)*		0.34 (0.05–2.26)
	male	4 (21)	5 (26.3)				5 (26.3)	4 (21)			
**Fluoride concentration in water considering gender**											
more than 1.5 ppm	female	123 (37.5)	45 (13.7)	0.333 *(c)*		1.28 (0.79–2.06)	141 (42.9)	27 (8.2)	0.054 *(c)*	0.88	1.74 (1.01–3) *
	male	109 (33.2)	51 (15.5)				120 (36.5)	40 (12.1)			
up to 1.5 ppm	female	171 (37.4)	124 (27.1)	0.628 *(c)*		1.1 (0.75–1.62)	209 (45.7)	86 (18.8)	0.171 *(c)*		1.36 (0.9–2.04)
	male	90 (19.6)	72 (15.7)				104 (22.7)	58 (12.6)			

* Significant association. *(a)* Dean and Thylstrup Fejerskov values were dichotomized for odds ratio (OR) and Fisher’s exact test; *(b) p* value by Person Chi-square test; *(c) p* value by Fisher’s exact test; *(d)* OR = odds ratio with 95% confidence interval (CI). **normal + QVM** = Dean index normal–questionable–very mild-mild; **MS** = Dean index moderate–severe; **normal + 1**–**3** = Thylstrup and Fejerskov Index normal plus levels 1, 2, and 3; **4**–**9** = Thylstrup and Fejerskov Index levels 4–9; **ppm** = part per million fluorides; **SP** = statistical power.
